# An unknown hotspot of plant diversity in the heart of the Central Apennine: flora and vegetation outline of Mt. Pozzoni-St. Rufo valley (Cittareale, Rieti)

**DOI:** 10.3897/phytokeys.178.62947

**Published:** 2021-05-31

**Authors:** Edda Lattanzi, Eva Del Vico, Roberto Tranquilli, Emmanuele Farris, Michela Marignani, Leonardo Rosati

**Affiliations:** 1 Via V. Cerulli 59, 00143 Roma, Italy Unaffiliated Rome Italy; 2 Dipartimento di Biologia Ambientale, Sapienza Università di Roma, P.le A. Moro 5, 00185 Roma, Italy Sapienza Università di Roma Rome Italy; 3 Via Achille Mauri 11, 00135 Roma, Italy Unaffiliated Roma Italy; 4 Department of Chemistry and Pharmacy, University of Sassari, Via Piandanna 4, 07100 Sassari, Italy University of Sassari Sassari Italy; 5 Department of Life and Environmental Sciences – Botany Division, University of Cagliari, Via Sant’Ignazio da Laconi 13, 09123 Cagliari, Italy University of Cagliari Cagliari Italy; 6 School of Agriculture, Forestry, Food and Environment, Via dell’Ateneo Lucano 10, University of Basilicata, 85100 Potenza, Italy University of Basilicata Potenza Italy

**Keywords:** endemic species, floristic records, Italy, phytosociology, Red lists

## Abstract

Surprisingly enough, Italy still has some botanically unexplored areas; among these there are some territories between Lazio, Umbria and Abruzzo not included in any protected area. The study area, ranging for 340 ha, includes the mountainous area of Mt. Pozzoni-Mt. Prato-St. Rufo valley, which forms the upper part of the river Velino basin, located in the territory of the municipality of Cittareale (Rieti, Lazio), at an elevation from 1150 to 1903 m a.s.l. The substrate is mainly made of marly limestone of the Meso-Cenozoic Umbria-Marche sedimentary succession. The climate is Temperate and comprises vegetation belts from the montane to sub-alpine. Land cover is dominated by pastures and deciduous forests, with only a few hay meadows. 794 entities have been detected: 16% are considered rare or very rare for the regional territory with several floristic novelties for the regional flora, 6% of the total was found to be endemic to Italy and only eight *taxa* were aliens. Four *taxa* are new for the regional flora of Lazio: *Arum cylindraceum*, *Alopecurus pratensis* subsp. *pratensis*, *Hieracium bupleuroides* and *Trinia glauca* subsp. *glauca.* Forest vegetation is represented by beech forests, while dry grasslands are the most widespread vegetation type. The greatest phytocoenotic diversity was found within the secondary pastures. Particularly interesting is the plant community with *Iris marsica*, which suggests that limestone mountain ledges can represent a primary habitat for this endemic species of the Central Apennine. The presence of several habitats listed in the EU Habitat Directive indicates how the lack of detailed territorial knowledge can lead to the non-designation of conservation sites in areas of high naturalistic value. These findings showed that botanical explorations in territories which are still not known could contribute significantly to the identification of areas of high interest in conserving plant diversity.

## Introduction

The study of Central Apennine attracted the attention of several botanists in the past (e.g. Gravina 1812; Tenore 1830; Paolucci 1891; Crugnola 1900; Grande 1904; Zodda 1931, 1954; Anzalone 1951; Montelucci 1952, 1953) for the presence of the highest peaks of the Italian peninsula and of a rich flora, characterized by the presence of numerous endemics (Conti 2004). The floristic knowledge of this territory has since been progressively increased by numerous contributions (e.g. Conti 1998, 2004; Ballelli 2003; Tondi et al. 2003; Di Pietro et al. 2008; Iocchi et al. 2010; Gubellini et al. 2014; Falcinelli et al. 2016; Conti et al. 2018; Rosati et al. 2020) and some synopses have recently involved the National Parks of the Central Apennine (Conti and Bartolucci 2015, 2016; Conti et al. 2019). On a regional scale, the state of floristic knowledge of this territory has been synthesized in the recent checklist of Italian vascular flora by Conti et al. (2005) and Bartolucci et al. (2018) whilst, for Lazio, a detailed flora was published by Anzalone et al. (2010). Despite this, as already highlighted in previous publications (Scoppola and Blasi 2005; Bartolucci et al. 2012, 2019), floristic exploration of several areas of the Central Apennine cannot be considered exhaustive and homogeneous throughout the territory; consequently, particularly interesting species, of high phytogeographic interest, are still being discovered (e.g. Cancellieri et al. 2017; Filibeck et al. 2020)

As for interior areas of the Central Apennine, the attention of botanists has always been directed towards the main mountain ranges (e.g. Terminillo, Sibillini, Laga), thus large portions of the surrounding territory have been neglected, both by floristic and vegetational studies. This is the case with the area constituting the upland drainage basin of Velino River, located between Lazio, Umbria and Abruzzo administrative regions where only wet meadows were studied (Venanzoni 1992). Some studies on a national scale had already highlighted that this area lacked specific floristic knowledge; in particular the Map of the Important Plant Areas in Italy (Blasi et al. 2009, 2011) indicated how this part of the Central Apennine is surrounded by areas of high interest for plant conservation, suggesting that covering such a gap of knowledge (Hortal et al. 2015) could lead to interesting results for its flora and vegetation.

In this study we present the results of the flora and vegetation surveys conducted by the authors during the period 2008–2010 in the mountainous area of Mt. Pozzoni-Mt. Prato-St. Rufo valley, which represents the upland drainage basin of the Velino River.

## Data and methods

### Study area

**Figure 1.**
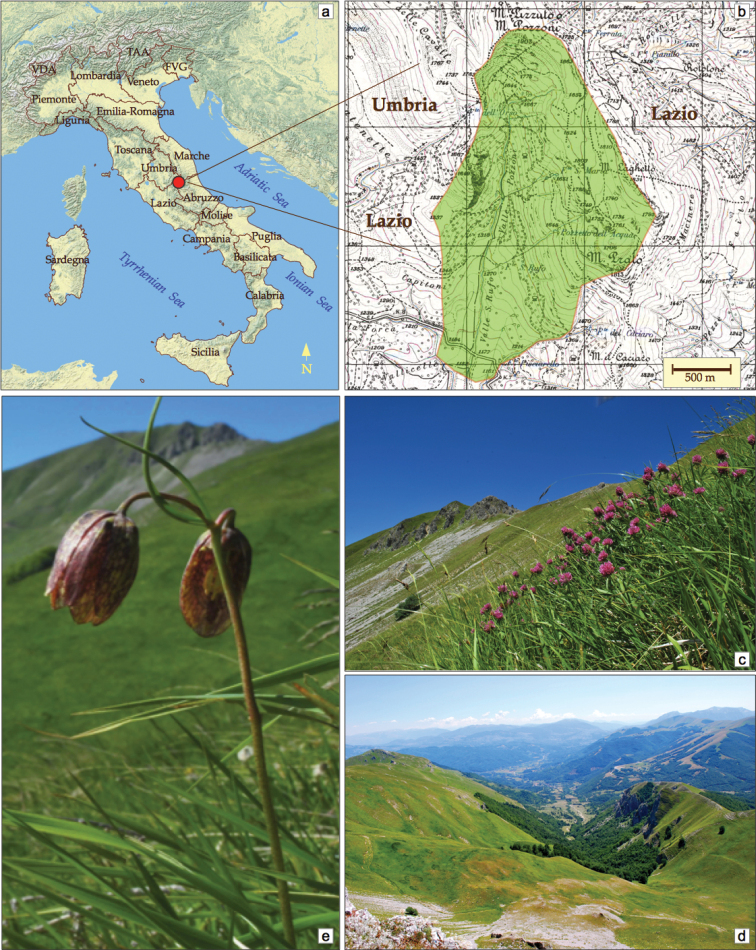
Study area location (**a**) and its landscape (**c–e**) **b** the limits of study area drawn on I.G.M.I-1:25,000 topographic map **c** high mountain pastures of Mt. Pozzoni dominated by *Brachypodium genuense* (DC.) Roem. et Schult. with *Trifolium alpestre* L. **e***Fritillaria montana* Hoppe ex W.D.J.Koch, a rare species considered “Near Threatened” for extinction risk. Photos: R. Tranquilli and E. Del Vico.

The study area is included within the municipality of Cittareale (province of Rieti), belonging to the Lazio administrative region. It extends for 343 ha, at altitudes ranging between 1150 and 1903 m a.s.l. (Fig. 1). The study area includes the peak of Mt. Pozzoni (1903 m), extends to southeast including the mountain ridge of Mt. Laghetto-Mt. Prato (1834 m), ending in the south almost in correspondence of the provincial road Cittareale-Norcia. To the west the limit runs along the watershed that delimits the valley of St. Rufo-Pozzoni (Fig. 1).

The substrate is mainly made up of Meso-Cenozoic marly limestone (“Scaglia” formation) belonging to the Umbria-Marche succession; these sedimentary layers are heavily fractured and faulted, due to the proximity of a regional overthrust overlapping the Sibillini unit on the Gran Sasso-Cittareale unit (Calamita et al. 1995). The head of the valley is modeled by an evident glacial cirque, with a threshold placed at an altitude of 1660 m; the valley bottom is largely covered with slope debris, mixed with sediments of fluvio-glacial origin partly terraced and dissected by the upper course of Velino River. Along St. Rufo valley, an important karst cavity, 3 km long and over 400 m deep, opens at about 1440 m set in the marly limestones of Scaglia rossa formation (Gatti and Uffreduzzi 1989); the cavity has returned interesting fossil remains of bats, testifying to a cold period fauna dated to the end of the Pleistocene (Argenti et al. 2008).

Following the bioclimatic classification of Rivas-Martínez et al. (2011), the climate is Temperate oceanic/semi-continental, with the presence of two phytoclimatic belts along the altitudinal gradient, from the lower supratemperate to the lower orotemperate of the cacuminal areas; ombrotypes are comprised between humid and hyperhumid (Pesaresi et al. 2017).

The land cover is dominated by secondary grasslands, deciduous woodlands and small patches of artificial coniferous forest. Meadows and fallows are very limited and arable lands are nowadays completely missing in the study area. In the past they must have been quite common in the lower part of the valley, as evidenced by the presence of several still visible terraced parcels. Forests are used regularly as coppices and summer grazing of cattle and sheep is still widespread in this sector, together with horse grazing, which is conversely continuously present almost all year round.

The study area does not comprise any protected area, even if some Natura 2000 sites, defined according to European Union Habitat Directive (European Union 1992), are present in the neighboring territory of the Umbria region.

### Flora and vegetation survey

The flora of the studied area was investigated in depth by carrying out numerous herborizations and field excursions, both in spring, summer and autumn, during three consecutive years from 2008 to 2010. Identification of vascular plants was mostly based on Pignatti (1982) and Flora Europaea (Tutin et al. 1968, 1972, 1976, 1980, 1993). *Taxa* delimitation was based on Anzalone et al. (2010) and nomenclature accords to Bartolucci et al. (2018), Galasso et al. (2018) and the subsequent updates summarized in the “Portal to the Flora of Italy” (http://dryades.units.it/floritaly/). Families of vascular plants correspond to APG IV (2016), whereas life forms and chorotypes were retrieved from Pignatti (1982). Exsiccata are preserved in *Herbarium Lucanum* (HLUC), *Herbarium Del Vico* (Roma) and *Herbarium Lattanzi* (Roma), the latter is now moving to *Herbarium Sapienza* (RO). In the floristic list we reported the rarity level in the regional flora of Lazio for each *taxon* according to Anzalone et al. (2010), adopting three levels: rare, medium rare and very rare (coded as R, MR, RR). New *taxa* for the regional flora were marked with an asterisk. Italian endemics were retrieved from Bartolucci et al. (2018). As for alien *taxa*, we also reported the status of naturalization in the study area following the same codes used by Galasso et al. (2018). For each *taxon* the status of threatened species was derived by the published Italian Red lists (Conti et al. 1992, 1997) and updated, when new assessments were available, according to the most recent ones (Rossi et al. 2013; Orsenigo et al. 2020). Vegetation was analyzed using the phytosociological method (Braun-Blanquet 1965) by carrying out 30 surveys of the main vegetation types in the territory located in the field with a GPS unit with ± 5 m accuracy. The syntaxonomic nomenclature, at the level of alliance, order and class, follows the ‘Prodrome of the Italian Vegetation’ (Biondi et al. 2014).

Main vegetation types were identified through multivariate methods, Hierarchical Cluster Analysis and NMDS ordination. For each vegetation type a floristic-ecological description and the syntaxonomic framework were provided.

## Results

### Flora

**Figure 2.**
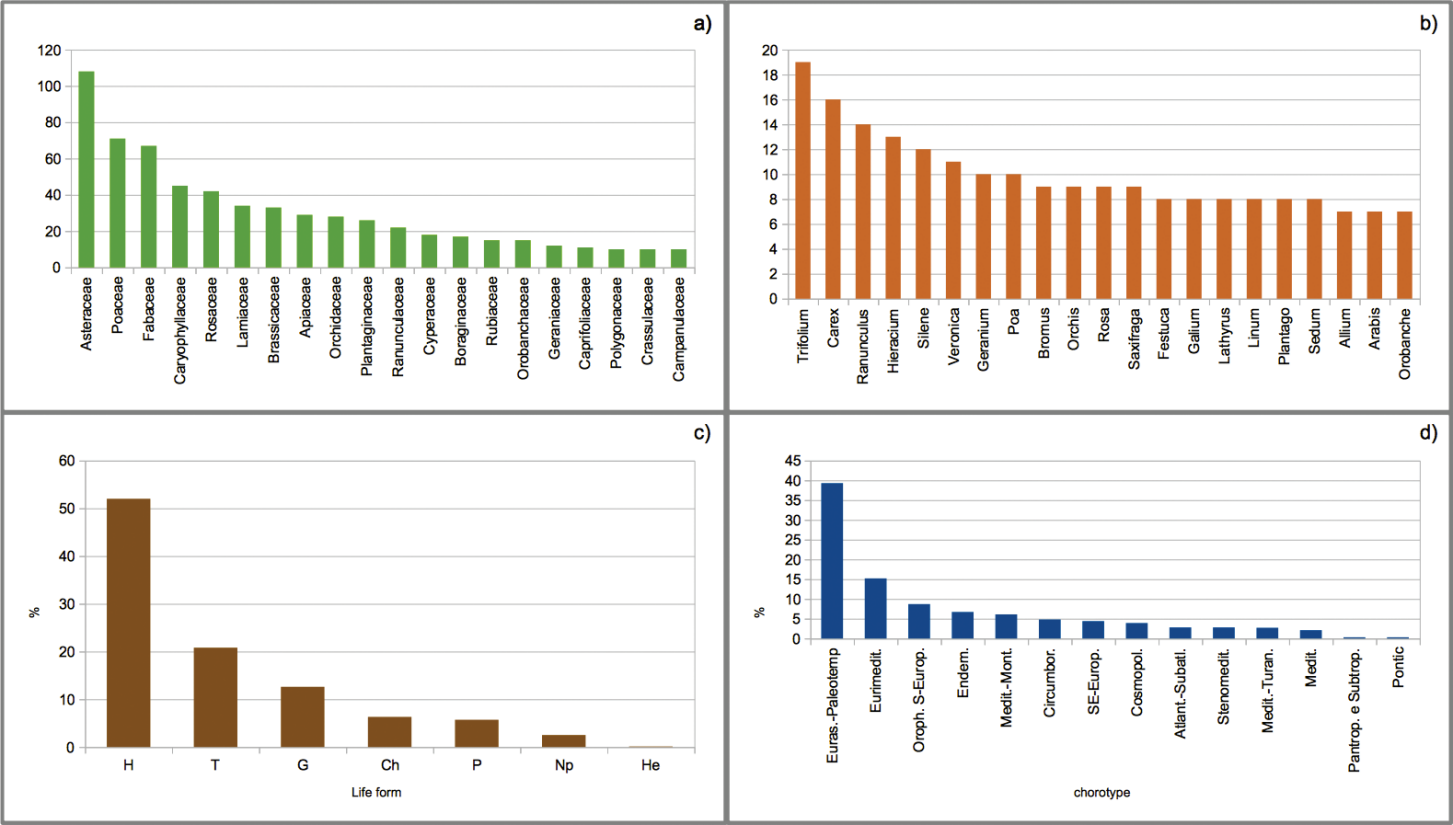
**a** number of *taxa* per family; only the families with more than 10 *taxa* are shown **b** number of *taxa* per genus; genera containing more than 7 *taxa* are shown **c** life form percentages **d** chorological spectrum of the detected flora in the study area.

794 *taxa* of vascular plants were identified, belonging to 331 genera and 69 families (Appendix 1). The families with more than 40 *taxa* (Fig. 2a) were: Asteraceae (107), Poaceae (71), Fabaceae (67), Caryophyllaceae (46) and Rosaceae (42). The most diverse genera were *Trifolium* (19), *Carex* (16), *Ranunculus* (14), *Hieracium* (13) and *Silene* (12) (Fig. 2b). Hemicryptophytes were the dominant life form (52%), followed by therophytes (21%) and geophytes (13%) (Fig. 2c). As for chorology, (Fig. 2d) species with Eurasian-Paleotemperate distribution prevailed (39%), slightly exceeding the Mediterranean element (29%). The Mediterranean group was mainly composed of Eurimediterranean (15%) and Mediterranean-Mountain species (6%). A significant contingent of Circumboreal species was also present (6%) while only a few species displayed eastern chorotypes (e.g. SE-European and Pontic).

Forty-seven *taxa*, approximately 6% of the total, were found to be endemic to Italy (Table 1). Only eight *taxa* were found to be aliens, among these *Malus domestica* (Suckow) Borkh., *Matricaria discoidea* DC., *Medicago sativa* L. and *Veronica persica* Poir. can be considered naturalized, whereas *Abies alba* Mill., *Abies cephalonica* Loudon, *Picea abies* (L.) H. Karst. and *Pinus nigra* J.F. Arnold subsp. *nigra* have been observed only as planted in reforested areas.

**Table 1.** List of endemic *taxa* recorded in the study area.

Taxon
*Armeria canescens* (Host) Ebel
*Astragalus sirinicus* Ten.
*Betonica alopecuros* L. subsp. *divulsa* (Ten.) Bartolucci & Peruzzi
*Brachypodium genuense* (DC.) Roem. et Schult.
*Campanula tanfanii* Podlech
*Carduus nutans* L. subsp. *perspinosus* (Fiori) Arènes
*Centaurea ambigua* Guss. subsp. *ambigua*
*Centaurea ambigua* Guss. subsp. *nigra* (Fiori) Pignatti
*Cerastium tomentosum* L.
*Corydalis densiflora* C.Presl subsp. *apennina* F.Conti, Bartolucci & Uzunov
*Crepis lacera* Ten.
*Cynoglossum apenninus* L.
*Cynoglossum magellense* Ten.
*Digitalis micrantha* Roth ex Schweigg.
*Epipactis meridionalis* H. Baumann et R. Lorenz
*Erysimum majellense* Polatschek
*Erysimum pseudorhaeticum* Polatschek
*Galium magellense* Ten.
*Gentianella columnae* (Ten.) Holub
*Helictochloa praetutiana* (Parl. ex Arcang.) Bartolucci, F.Conti, Peruzzi & Banfi subsp. *praetutiana*
*Iris marsica* I. Ricci et Colas.
*Klasea flavescens* (L.) Holub subsp. *cichoracea* (L.) Greuter et Wagenitz
*Koeleria splendens* C. Presl
*Linaria purpurea* (L.) Mill.
*Myosotis decumbens* Host subsp. *florentina* Grau
*Myosotis* graui Selvi
*Ornithogalum etruscum* Parl.
*Oxytropis pilosa* (L.) DC. subsp. *caputoi* (Moraldo et La Valva) Brilli-Catt., Di Massimo et Gubellini
*Pedicularis elegans* Ten.
*Potentilla rigoana* Th.Wolf
*Pulmonaria vallarsae A.Kern.* subsp. *apennina* (Cristof. & Puppi) L.Cecchi & Selvi
*Ranunculus apenninus* (Chiov.) Pignatti
*Ranunculus thomasii* Ten.
*Rhinanthus wettsteinii* (Sterneck) Soó
*Saxifraga exarata* Vill. subsp. *ampullacea* (Ten.) D.A.Webb
*Saxifraga porophylla* Bertol. subsp. *porophylla*
*Sempervivum riccii* Iberite et Anzal.
*Senecio apenninus* Tausch
*Sesleria nitida* Ten
*Silene notarisii* Ces.
*Siler montanum* Crantz subsp. *siculum* (Spreng.) Iamonico, Bartolucci & F.Conti
*Stipa dasyvaginata* Martinovský subsp. *apenninicola* Martinovský et Moraldo
*Trifolium pratense* L. subsp. *semipurpureum* (Strobl) Pignatti
*Trisetaria villosa* (Bertol.) Banfi et Soldano
*Viola eugeniae* Parl. subsp. *eugeniae*

Two taxa, *Arum cylindraceum* Gasp. and *Corydalis densiflora* C.Presl subsp. *apennina* F.Conti, Bartolucci & Uzunov have been recorded for the first time for Lazio during this research, whereas four *taxa* whose presence was considered doubtful for Lazio have been confirmed (*Alopecurus pratensis* L. subsp. *pratensis*, *Hieracium bupleuroides* C.C.Gmel., *Scandix macrorhyncha* C.A.Mey and *Trisetaria flavescens* (L.) Baumg. subsp. *flavescens*). These floristic records at regional level have been anticipated by Del Vico et al. (2014). However, probably due to oversight, *Alopecurus pratensis* subsp. *pratensis* is still considered to be confirmed for Lazio in the continuously updated database of the Portal to the Flora of Italy (Available at http:/dryades.units.it/floritaly, accessed: 16/11/2020). Additionally, we herein confirm the presence of *Trinia glauca* (L.) Dumort. subsp. *glauca*, previously considered recorded erroneously for Lazio (Bartolucci et al. 2018).

Thirty-four *taxa* (Table 2) are considered very rare at the regional level (2010). Considering all the rare species (R, MR and RR), these *taxa* represent altogether approximately 20% of the studied flora.

**Table 2.** List of *taxa* found in the study area considered very rare (RR) at regional level including those that are new records for Lazio with respect to Anzalone et al. (2010).

Taxon
*Achillea tomentosa* L.
*Alchemilla cinerea* Buser
*Arabis auriculata* Lam.
*Arum cylindraceum* Gasp.
*Avenella flexuosa* (L.) Drejer subsp. *flexuosa*
*Carex liparocarpos* Gaudin subsp. *liparocarpos*
*Carex panicea* L.
*Centaurea arachnoidea* subsp. *adonidifolia* (Rchb.) F. Conti, Moraldo & Ricceri
*Conringia austriaca* (Jacq.) Sweet
*Corydalis densiflora* C.Presl subsp. *apennina* F.Conti, Bartolucci & Uzunov
*Epipactis meridionalis* H. Baumann et R. Lorenz
*Erysimum majellense* Polatschek
*Gagea minima* (L.) Ker Gawl.
*Genista sagittalis* L.
*Herniaria glabra* L. subsp. *nebrodensis* Nyman
*Hieracium tomentosum* L.
*Hypericum hyssopifolium* Chaix
*Iris marsica* I. Ricci et Colas.
*Juncus striatus* Schousb. ex E. Mey.
*Mcneillia graminifolia* (Ard.) Dillenb. & Kadereit subsp. *clandestina* (Port.) Dillenb. & Kadereit
*Medicago prostrata* Jacq. subsp. *prostrata*
*Onobrychis arenaria* (Kit.) DC. subsp. *arenaria*
*Oxytropis pilosa* (L.) DC. subsp. *caputoi* (Moraldo et La Valva) Brilli-Catt., Di Massimo et Gubellini
*Parnassia palustris* L. subsp. *palustris*
*Pilosella cymosa* (L.) F.W.Schultz & Sch.Bip.
*Pilosella hoppeana* (Schult.) F.W.Schultz & Sch.Bip.
*Pilosella piloselloides* (Vill.) Soják subsp. *praealta* (Vill. ex Gochnat) S.Bräut. & Greuter
*Scorzonera laciniata* L.
*Scorzonera purpurea* L. subsp. *purpurea*
*Thymus oenipontanus* Heinr.Braun ex Borbás
*Trifolium phleoides* Willd.
*Trinia glauca* (L.) Dumort. subsp. *glauca*
*Trisetaria villosa* (Bertol.) Banfi et Soldano
*Tulipa pumila* Moench

The following 15 species have been identified as being at risk of extinction in the first published Italian red lists (Conti et al. 1992, 1997): *Iris marsica* I. Ricci et Colas., *Achillea tomentosa* L., *Carex panicea* L., *Fritillaria montana* Hoppe ex Koch, *Gentiana lutea* L. subsp. *lutea*, *Gentiana utriculosa* L., *Klasea nudicaulis* (L.) Fourr., *Lathyrus nissolia* L., *Lilium bulbiferum* L. subsp. *croceum* (Chaix) Jan, *Narcissus poëticus* L., *Onobrychis arenaria* (Kit.) DC. subsp. *arenaria*, *Ornithogalum comosum* L., *Scorzonera purpurea* L., *Trifolium phleoides* Willd. and *Trisetaria villosa* (Bertol.) Banfi et Soldano. Meanwhile, in the most recent ones (Rossi et al. 2013; Orsenigo et al. 2020), excluding the species classified as “Least Concern”, *Viola kitaibeliana* Schult. was listed as “Endangered”, *Epipactis meridionalis* H. Baumann et R. Lorenz as “Vulnerable” and *Fritillaria montana*, *Gentiana lutea*, *Iris marsica* and *Senecio scopolii* Hoppe et Hornsch. ex Bluff et Fingerh. as “Near Threatened”.

### Plant community descriptions

**Figure 3.**
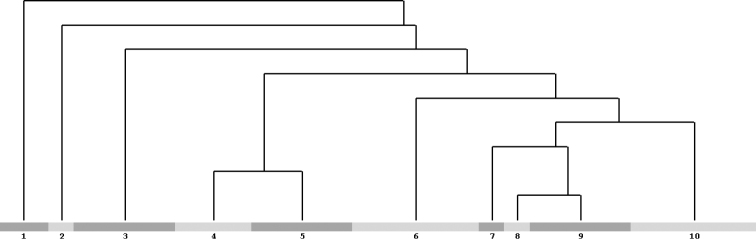
Hierarchical clustering resulting from Modified TWINSPAN analysis. Cluster 1) *Fagus sylvatica* woodlands; 2) *Amelanchier ovalis* forest edges; 3) *Saxifraga callosa* chasmophytic vegetation; 4) *Cynosurus cristatus* hay meadows; 5) *Bromopsis erecta* montane grasslands; 6) *Iris marsica* community; 7) *Rumex scutatus* screes vegetation; 8) *Paronychia kapela* rocky outcrops vegetation; 9) upper-montane grasslands, including also the *Brachypodium genuense* community and the *Astragalus sempervirens* community; 10) chamaephytes-rich dry grasslands.

Hierarchical cluster analysis and NMDS ordination (the latter not shown) enabled the detection of 10 clusters that were clearly interpretable, floristically and ecologically, as shown in Fig. 3. A further inspection of the ordered table led to the identification of 12 plant communities (some of these represented by only one relevè); the greatest phytocoenotic diversity was found within the secondary pastures. The relevès of these communities are presented in Suppl. material 1: Table S1 and described in detail below.

### Forests and shrublands

Forest vegetation in the Mt. Pozzoni valley is represented almost exclusively by beech forests (cluster 1). *Quercus cerris* woodlands are present only on the edges of the investigated area on southern exposures, but they are widespread at lower altitudes where flyschoid substrates emerge. Small patches of coniferous plantation, planted for reforestation purposes (e.g. *Pinus nigra*, *Abies spp.*) are also present. *Fagus sylvatica* forests cover approx. 60 ha, forming a more or less continuous belt between 1350 and 1700 m a.s.l.; they are managed as coppices with stands, meanwhile mixed aged and old-growth formations are absent. Tree layer is always dominated by *Fagus sylvatica*, frequently accompanied by *Acer pseudoplatanus* and *Acer opalus* subsp. *obtusatum*. In relevè no. 1, surveyed at 1500 m, in the tree layer we also found *Tilia plathyphyllos* and *Prunus avium*, while in rel. no. 2, placed at 1360 m, the presence of *Quercus cerris* and *Acer campestre* can be noted. In the herb layer, nemoral species commonly found in mesic and beech forests are present, such as *Cardamine bulbifera*, *Pulmonaria vallarsae* subsp. *apennina*, *Moehringia trinervia*, *Rabelera holostea* (= *Stellaria holostea*); from a phytosociological point of view most of these species are typical of *Fagetalia sylvaticae* order. Related to the altitude, the second relevè in table S1 hosts a larger contingent of species having a relatively thermophilic character, such as *Lathyrus venetus*, *Cyclamen hederifolium*, *Cephalanthera damasonium*, *Sesleria autumnalis*, *Viola odorata*, which also characterize the lower altitudinal vegetation belt dominated by oak forests. These relevès can be classified in the alliance *Aremonio agrimonioidis-Fagion sylvaticae* (*Querco roboris-Fagetea sylvaticae*, *Fagetalia sylvaticae*) describing the beech forests of E-Europe, Balkans and extending to Central-Northern Apennine (Biondi et al. 2014). The beech woods at lower altitudes, as evidenced by the relevè no. 2, show a floristic composition referable to *Lathyro veneti-Fagetum sylvaticae*, a widespread association which frames beech forests of lower supratemperate belt of the Central Apennines mainly on limestones (Blasi et al. 2010). At higher altitudes, generally above 1500 m, it is possible to observe a clear decrease in the number of species of *Quecetalia pubescenti-petraeae*, indicating a shift towards the association *Cardamino kitaibelii-Fagetum sylvaticae* Ubaldi ex Ubaldi 1995. The latter represents the potential vegetation of the upper supratemperate belt, which in the St. Rufo valley is almost completely substituted by the secondary grasslands belonging to *Festuco-Brometea* class.

The edge of the beech forest (cluster 2), in some cases, presents characteristic pre-forest shrub formations, as evidenced by relevè no. 3, physiognomically dominated by *Amelanchier ovalis*, *Atadinus fallax* (=*Rhamnus alpina* subsp. *fallax*) and *Sorbus aria*, accompanied by smaller shrubs of *Rosa montana*, *Crataegus laevigata* and *Cytisophyllum sessilifolium.* These formations can be attributed to the association *Rhamno alpinae-Amelanchieretum ovalis* (*Berberidion vulgaris*, *Prunetalia spinosae*, *Rhamno-Prunetea*), described for Sibillini mountains by Pedrotti (1994) and revised by Cutini et al. (2002), even if the herb layer appeared strongly impoverished.

Along the river incisions, especially in the middle and lower part of the investigated area, there are other shrub and pre-forest formations of difficult syntaxonomic classification at association level, dominated by *Corylus avellana* and *Acer spp*., which are not represented in our surveys.

### Sparse vegetation of outcrops

Limestone rocky walls, from 1400 m up to the highest peak of Mt. Pozzoni, are colonized by a characteristic chasmophytic vegetation dominated by *Saxifraga callosa* (rel. 4–6) with the presence of *Saxifraga paniculata*, *Campanula tanfanii* and *Saxifraga exarata* subsp. *ampullacea* (cluster 3). Even if *Trisetum villosum* was not found within the relevès, this species is present in such habitats of the study area (see floristic list). The overall floristic composition of these coenoses allows us to refer them to *Saxifrago australis*-*Trisetetum bertolonii* (*Saxifragion lingulatae*, *Potentilletalia caulescentis*, *Asplenietea trichomanis*), a widespread association in the Umbria-Marche Apennine and Central Italy (Giancola and Stanisci 2002).

On sub-vertical calcareous rocky outcrops, large boulders and ledges, we found some peculiar communities characterized by the abundance and dominance of *Sedum album*. At higher altitudes, above 1650 m (rel. no. 7–9) these coenoses are characterized by *Paronychia kapela*, *Anthemis cretica* subsp. *columnae*, *Sempervivum arachnoideum* and *Poa molinerii* (cluster 8). The floristic composition is dominated by species belonging to *Sedo albi-Scleranthetea biennis* such as *Sedum album*, *Sempervivum arachnoideum* and *Petrosedum rupestre* together with several transgressive species from dry grasslands of *Festuco-Brometea*. These coenoses developed on a calcareous substrate have to be attributed to the alliance *Alysso alyssoidis-Sedion albi.* At the alliance level, the same attribution is valid for the relevès no.10–12 (cluster 6), carried out on large rocky ledges at lower altitudes (ca 1500 m) which are physiognomically characterized by the endemic *Iris marsica* and by *Petrosedum rupestre*, with the presence of several thermophilous species such as *Crupina vulgaris*, *Lactuca perennis* subsp. *perennis* and *Centaurea deusta.* The survey no. 13, characterized also by the presence of *Iris marsica* but with the dominance of *Bromopsis erecta*, represents the ecotone between the vegetation of the *Alysso-Sedion* on ledges and the contiguous dry grasslands of *Festuco-Brometea*, which are developed where the soils are more evolved and deeper. Our current state of knowledge about *Alysso-Sedion* vegetation in the studied area does not make it possible to find a clear reference to any already described association; therefore these two coenoses are provisionally indicated as *Sedum album-Paronychia kapela* community and *Sedum album*-*Iris marsica* community.

Calcareous screes and mobile debris have only small surfaces in the study area; they are represented by the relevè no. 14 (cluster 7), carried out at medium altitude (1480 m), on the scree fed by the characteristic rocky spike present on the right side of the St. Rufo valley, known as Peschio dell’Aquila. This plant community has sparse cover and is characterized by *Rumex scutatus*, *Galium magellense* and *Scrophularia canina.* The scree vegetation of the alpine and subalpine belt in the Central Apennine is quite well known, while at lower altitudes it has rarely been investigated (see Ciaschetti et al. 2020 and references therein). Also in this case, it was not possible to identify a reference at the association level for the *Petrosedum rupestre-Rumex scutatus* community of the study area. The presence of *Galium magellense* suggests a probable framework within the alliance *Linario-Festucion dimorphae*, describing Apennine glareicolous communities of calcareous screes, from the supratemperate to orotemperate thermotypes, even if most of the diagnostic species are lacking. The floristic impoverishment could be justified by the fact that in the study area the most important screes are located at rather low altitudes, at the lower ecological limit of *Linario-Festucion dimorphae.* In fact, at higher altitude we observed in this habitat also the presence of *Drypis spinosa.* Although the abundance of *Petrosedum rupestre* would seem to indicate an affinity with the association *Linario purpureae-Petrosedetum rupestris* recently described for several localities of the Abruzzo territory (Ciaschetti et al. 2020), the overall floristic composition of the community herein reported is rather different from the one described for Abruzzo and the diagnostic species are absent. In addition, this thermophilic association was referred to a different order and alliance (*Scrophulario bicoloris-Helicrhysetalia italici* Brullo, 1984, *Linarion purpureae* Brullo, 1984) within the class *Thlaspietea rotundifolii*.

### Meadows and grasslands

In the lower part of the study area, at an altitude below 1200 m, small patches of regularly mowed grasslands are still present (cluster 4; rel. no. 15–17), developed on deep, partially terraced, well drained alluvial soil. They are species rich, with 54 species per plot on average. Floristically, the dominant species (*Trifolium incarnatum*, *T. repens*, *T. pratense*, *Poa trivialis*, *Dactylis glomerata*, *Phleum nodosum*, *Cynosurus cristatus*) are indicators of the class *Molinio-Arrhenatheretea*, accompanied by several nitrophilous and ruderal species such as *Bromus hordeaceus* and *Dasypyrum villosum.* The perennial meso and supratemperate Apennine grasslands, usually grazed and mowed once a year, which develop on mesotrophic soils with good water availability and rich in nutrients, have been commonly attributed to the *Cynosurion cristati* alliance, (see the Italian review of the alliance in Blasi et al. 2012 and references therein). The classification of *Cynosurion* grasslands has often been considered to be somewhat problematic (Blasi et al. 2012) due to the fact that exclusive species are lacking and most of the diagnostic species range widely from both a geographical and ecological point of view. Although the surveys carried out in the study area can be referred to *Cerastio arvensis-Cynosurenion cristati* sub-alliance, the attribution to an already described association remains unresolved, also because the Apennine’s associations have not yet been the subject of a critical review which clarifies delimitation and differential species. Probably the closest association could be identified in the *Colchico lusitani-Cynosuretum*, repeatedly reported on the Umbria-Marche Apennines (e.g. Allegrezza 2003; Biondi et al. 2004; Catorci et al. 2007).

The most widespread vegetation in the study area is represented by the dry grasslands of the *Festuco-Brometea* class (cluster 5; rel. no. 18–30), mainly used for horse and cattle grazing, which replaced the beech forest vegetation on the mountain slopes. In particular, they can be classified in *Phleo ambigui-Brometalia* order including xerophilous and semi-mesophilous secondary grasslands of the Central-southern Apennines, that occur from the supramediterranean to the upper supratemperate thermotype. These plant communities have a high physiognomic and floristic diversification in relation to local characteristics of the site (exposure, rockiness, soil depth and pH) and to the degree of grazing. The relevès no. 18–22 (cluster 10) are particularly species rich (mean 52 species per releve) and were found at lower altitude (max 1300 m), frequently on moraine deposits. They are characterized by a high incidence of chamaephytes (e.g. *Helianthemum apenninum*, *H. oelandicum* subsp. *incanum*, *H. nummularium* subsp. *obscurum*, *Thymus longicaulis*, *T. oenipontanus*) and by the presence of several therophytes (e.g. *Trifolium campestre*, *T. scabrum*, *Euphrasia liburnica*). However, they are physiognomically dominated by hemicryptophytes such as *Bromopsis erecta*, *Festuca circummediterranea*, *Phleum hirsutum* subsp. *ambiguum*. From a phytosociological point of view, the ecology and floristic analysis led to the inclusion of this community in the association *Asperulo purpureae-Brometum erecti*, frequently reported for the Umbria-Marche Apennine (e.g. Catorci et al. 2007) and described with several variants and subassociations. The releve no. 23 represents a particular stand on strongly inclined slopes at higher altitude (1500 m) dominated by *Bromopsis erecta* but with a high cover of *Securigera varia* and *Petrosedum rupestre*, which cannot be easily classified at the level of association.

At higher altitude (cluster 9) and on more inclined slopes, in the dry grasslands can be noted the presence of *Sesleria nitida* and a floristic composition close to the association *Seslerio nitidae-Brometum erecti.* Near the summit area of the mountain slopes or in eroding areas, the floristic composition becomes impoverished and the spiny chamaephyte *Astragalus sempervirens* was found as co-dominant with *Sesleria nitida*. The reference for this community is the *Astragalo sempervirenti-Seslerietum nitidae*, an association described for the grasslands of the summit sectors of Coscerno and Civitella Mountains in Umbria (Biondi and Ballelli 1995). The parts at higher altitudes of the mountain slopes, relying on calcareous-marly substrata, are occupied by grasslands dominated by *Brachypodium genuense*. The presence of some acidophilic species (e.g. *Luzula campestris*, *Genista sagittalis*, *Campanula micrantha*) which are frequently found in the vegetation of *Nardetea strictae* (see Di Pietro et al. 2017 for a review of these communities in the Italian Peninsula), indicates the presence of decarbonated soils with a lower pH. Despite this, the floristic composition is clearly dominated by *Festuco-Brometea* species; therefore, the reference for these coenoses, is the order *Phleo ambigui-Brometalia erecti* and the alliance *Phleo-Bromion erecti*, but unlike the previous ones, in this case the arid high-montane grasslands are to be referred to the sub-alliance *Brachypodienion genuensis.* At the association level, the floristic analysis led to referring this community to the *Potentillo rigoanae-Brachypodietum genuensis*, an association quite widespread in the Central Apennine, in particular in Lazio and Abruzzo regions (Lucchese et al. 1995).

We did not survey other relevant coenoses that are present with significant extensions but only outside the surveyed area: the mountain acidophilic grasslands of the *Nardetea strictae* class, present on the northern slopes of Mt. Pozzoni and in the concave morphologies in the high-mountain orotemperate belt and the discontinuous prairies of the steep slopes dominated by *Sesleria juncifolia*; the latter can be observed on the steep and inaccessible mountain slope surrounding the cliff of Peschio dell’Aquila.

## Discussions and conclusions

The floristic composition of the study area, at the family level, does not significantly differ from the neighboring regional floras (Anzalone et al. 2010; Conti 1998). The percentage of endemics (6%) was quite similar to the flora of the nearby Terminillo massif (5.1%) studied by Montelucci (1952, 1953), slightly lower than the flora of Gran Sasso National Park (8.7%) (Conti and Bartolucci 2016), but perfectly aligned, for example, with the value of Abruzzo National Park (6.5%) (Conti and Bartolucci 2015). This difference can be due to the fact that the higher elevation of Gran Sasso National Park allows the presence of a large alpine vegetation belt, known to host several endemic *taxa*. On the contrary, in the study area, Terminillo massif and Abruzzo National Park, the alpine belt is completely absent. In fact, only considering the ipsophilous flora (above 1900 m a.s.l.), Conti (2004) calculated a rate of 13.2% of endemics for Central Apennine.

Similarly, from the chorogical point of view, no particular differences were observed with the flora of Terminillo, except for the contingent of Illirian, SE-European and Pontic species, which are slightly more represented in the flora of the Terminillo, probably because the flora of the latter also includes the lower vegetation belts of thermophilous oak forests and mixed deciduous woods, known to be characterized by the presence of numerous eastern species (Blasi et al. 2004).

Based on overall floristic results, the area of the St. Rufo Valley-Mt. Pozzoni can be considered of particular floristic interest, due to the high number of endemic and rare species detected, as recognized also by Lucchese (2018) who incorporated our preliminary data (Del Vico et al. 2014) in his recent, but not yet completed, atlas of the flora of Lazio.

The studied area is also undoubtedly characterized by a high floristic diversity if we consider that the 794 *taxa* recorded in this study have been found in an area of only 3,4 km^2^. The relevance of these data can be easily understood by comparing the number of *taxa* detected in local floras that have a comparable extension. For example, considering the data reported in Pierini et al. (2009), regarding numerous floras from Tuscany, and limiting to those having extension between 2 and 8 km^2^, only in the flora of Mt. Ferrato a similar number of *taxa* (800) was found, but within a study area almost double the size (6 km^2^) (Biagioli et al. 2002).

Moreover, we have to keep in mind that the studied area has a modest altitude range, relatively few types of lithologies and thus, a limited number of habitats. Despite this, the vegetation analysis revealed the presence of varied and species rich plant communities, with 311 *taxa* detected in only 30 relevès. As expected, the higher number of vegetation types was found within the secondary habitats of mountain pastures and dry grasslands. Particularly interesting is the plant community with *Iris marsica* (referred to the *Alysso-Sedion* alliance), which indicates, probably for the first time, as limestone mountain ledges can represent a primary habitat for this species endemic of the Central Apennine.

Almost all the plant communities identified here are referable to habitats listed by the Habitat Directive (habitat codes: 6110, 6210, 6520, 8120, 8210, 9210), some of them with priority status (6110, 6210, 9210), thus their presence would have required the proposal of a Natura 2000 site according to the Habitat Directive (European Union 1992).

The intriguing aspects of this territory combined with, until recently, the complete lack of detailed botanical knowledge, led, in 2016, the Italian Botanical Society to carry out, in the Cittareale municipality, an annual field trip of the working group for Floristics, Systematics and Evolution. During the field trip, with the participation of some of the authors, several other additional localities (e.g. Mt. Boragine 1824 m a.s.l.), surrounding those herein investigated, were explored floristically (Bartolucci et al. 2019).

The very limited number of alien species identified, none of which is considered invasive, can be considered an indicator of the fairly good state of conservation of the territory in which a completely traditional land use still persists. However, reforested areas, planted with several non- native conifers, are now composed of mature trees able to produce seeds. Recruitment from these could involve a process of spontaneization of non-native coniferous as frequently observed in other territories of Central Apennine, involving, for example, the spread of *Pinus nigra*. Moreover, the presence of *Abies cephalonica* and *Picea abies* in reforested areas could also lead to spontaneization of these species in Lazio, as already observed in the neighboring Abruzzo region (Galasso et al. 2018).

Taking into account how important updating distribution data is, for example for the Red List assessment (Orsenigo et al. 2020), it is certainly possible to affirm that this study constitutes a valid contribution towards filling the gap in our botanical knowledge of a sector of the Central Apennines of high conservation interest.

## Syntaxonomic scheme

**ASPLENIETEA TRICHOMANIS (Br.-Bl. in Meier & Br.-Bl., 1934) Oberdorfer, 1977**

POTENTILLETALIA CAULESCENTIS Br.-Bl. in Br.-Bl. & Jenny, 1926

Saxifragion australis Biondi & Ballelli ex Brullo 1984

*Saxifrago-Trisetetum villosi* Biondi & Ballelli, 1982

**THLASPIETEA ROTUNDIFOLII Br.-Bl., 1948**

THLASPIETALIA STYLOSI Avena & Bruno, 1975

Linarion purpureae Brullo, 1984

*Petrosedum rupestre-Rumex scutatus* community

**SEDO ALBI-SCLERANTHETEA BIENNIS Br.-Bl,. 1955**

ALYSSO ALYSSOIDIS-SEDETALIA ALBI Moravec, 1967

Alysso alyssoidis-Sedion albi Oberdorfer & Müller in Müller 1961

*Sedum album-Paronychia kapela* community

*Sedum album-Iris marsica* community

**FESTUCO VALESIACAE-BROMETEA ERECTI Br.-Bl. & Tüxen ex Br.-Bl. 1949**

PHLEO AMBIGUI-BROMETALIA ERECTI Biondi, Allegrezza, Blasi & Galdenzi in Biondi, Allegrezza, Casavecchia, Galdenzi, Gasparri, Pesaresi, Vagge and Blasi 2014

Phleo ambigui-Bromion erecti Biondi, Ballelli, Allegrezza & Zuccarello ex Biondi and Galdenzi 2012

Phleo ambigui-Bromenion erecti Biondi, Allegrezza & Zuccarello ex Di Pietro 2011

*Asperulo purpureae-Brometum erecti* Biondi & Ballelli ex Di Pietro 2011

*Seslerio nitidae-Brometum erecti* Bruno & Covarelli, 1968

*Astragalo sempervirentis-Seslerietum nitidae* Biondi & Ballelli, 1995

Brachypodienion genuensis Biondi, Ballelli, Allegrezza & Zuccarello ex Biondi and Galdenzi 2012

*Potentillo rigoanae-Brachypodietum genuensis* Lucchese et al., 1995

**MOLINIO-ARRHENATHERETEA Tüxen, 1937**

TRIFOLIO REPENTIS-PHLEETALIA PRATENSIS Passarge, 1969

Cynosurion cristati Tüxen, 1947

Cerastio arvensis-Cynosurenion cristati Blasi et al., 2012

*Trifolium incarnatum-Cynosurus cristatu*s community

**RHAMNO CATHARTICAE-PRUNETEA SPINOSAE Rivas Goday & Borja ex Tüxen 1962**

PRUNETALIA SPINOSAE Tüxen, 1952

Berberidion vulgaris Br.-Bl., 1950

*Rhamno alpinae-Amelanchieretum ovalis* Pedrotti, 1994

**QUERCO ROBORIS-FAGETEA SYLVATICAE Br.-Bl. & Vlieger in Vlieger 1937**

FAGETALIA SYLVATICAE Pawłowski in Pawłowski, Sokołowski and Wallisch 1928

Aremonio agrimonioidis-Fagion sylvaticae (Horvat) Borhidi in Török, Podani and Borhidi 1989

*Lathyro veneti-Fagetum sylvaticae* Biondi et al. ex Biondi, Casavecchia, Pinzi, Allegrezza and Baldoni in Biondi, Allegrezza, Casavecchia, Galdenzi, Gigante and Pesaresi 2013

## Appendix 1

**Table:** Floristic list of detected *taxa* in the study area. E = endemic *taxon* of Italian territory; R, MR, RR = increasing level of rarity, form rare to very rare in the regional flora of Lazio coded as in Anzalone et al. (2010). Floristic novelties for the regional flora are marked with asterisk. CULT = *taxon* detected only as cultivated. NAT = alien *taxon* naturalized in the study area.

Taxon	
**Ferns and ferns allies**	
Selaginellaceae	
	*Selaginella denticulata* (L.) Spring
Equisetaceae	
	*Equisetum palustre* L.
	*Equisetum ramosissimum* Desf.
	*Equisetum telmateja* Ehrh.
Ophioglossaceae	
R	*Botrychium lunaria* (L.) Sw.
Cystopteridaceae	
	*Cystopteris fragilis* (L.) Bernh.
Aspleniaceae	
	*Asplenium ceterach* L. subsp. *bivalens* (D.E.Mey.) Greuter & Burdet
	*Asplenium onopteris* L.
	*Asplenium ruta-muraria* L. subsp. *ruta-muraria*
	*Asplenium trichomanes* L. subsp. *quadrivalens* D.E. Mey.
**Gymnosperms**	
Pinaceae	
CULT	*Abies alba* Mill.
CULT	*Abies cephalonica* Loudon
CULT	*Picea abies* (L.) H. Karst.
CULT	*Pinus nigra* J.F. Arnold subsp. *nigra*
Cupressaceae	
	*Juniperus communis* L. subsp. *alpina* (Suter) Celak
	– *taxon* delimitation according to Anzalone et al. (2010)
	*Juniperus communis* L. subsp. *communis*
	– *taxon* delimitation according to Anzalone et al. (2010)
	*Juniperus oxycedrus* L. subsp. *oxycedrus*
	– *taxon* delimitation according to Anzalone et al. (2010)
**Angiosperms**	
Araceae	
*RR	*Arum cylindraceum* Gasp. ex Guss.
	*Arum italicum* Mill. subsp. *italicum* var. *italicum*
	*Arum maculatum* L.
Colchicaceae	
	*Colchicum lusitanum* Brot.
Liliaceae	
R	*Fritillaria montana* Hoppe ex W.D.J.Koch
	*Gagea lutea* (L.) Ker Gawl.
RR	*Gagea minima* (L.) Ker Gawl.
	*Gagea villosa* (M. Bieb.) Sweet
	*Lilium bulbiferum* L. subsp. *croceum* (Chaix) Jan
RR	*Tulipa pumila* Moench
Orchidaceae	
	*Anacamptis morio* (L.) R.M.Bateman, Pridgeon & M.W.Chase
	*Anacamptis pyramidalis* (L.) Rich.
	*Cephalanthera damasonium* (Mill.) Druce
	*Cephalanthera longifolia* (L.) Fritsch
	*Cephalanthera rubra* (L.) Rich.
	*Coeloglossum viride* (L.) Hartm.
	*Dactylorhiza maculata* (L.) Soó subsp. *saccifera* (Brongn.) Diklić
	*Dactylorhiza sambucina* (L.) Soó
	*Epipactis atrorubens* (Hoffm.) Besser
E – RR	*Epipactis meridionalis* H. Baumann & R. Lorenz
	*Epipactis microphylla* (Ehrh.) Sw.
	*Epipactis muelleri* Godfery
	*Gymnadenia conopsea* (L.) R. Br.
	*Himantoglossum adriaticum* H. Baumann
	*Neotinea tridentata* (Scop.) R.M.Bateman, Pridgeon & M.W.Chase
	*Neotinea ustulata* (L.) R.M.Bateman, Pridgeon & M.W.Chase
	*Neottia nidus-avis* (L.) Rich.
	*Neottia ovata* (L.) Bluff & Fingerh.
	*Ophrys apifera* Huds.
	*Ophrys holosericea* (Burnm.f.) Greuter subsp. *holosericea*
	*Ophrys sphegodes* Mill. subsp. *sphegodes*
	*Orchis anthropophora* (L.) All.
	*Orchis mascula* (L.) L. subsp. *mascula*
MR	*Orchis pallens* L.
	*Orchis pauciflora* Ten.
	*Orchis purpurea* Huds.
	*Orchis simia* Lam.
	*Platanthera bifolia* (L.) Rich.
Iridaceae	
	*Crocus vernus* (L.) Hill
E – RR	*Iris marsica* I. Ricci & Colas.
Asphodelaceae	
	*Asphodeline lutea* (L.) Rchb.
Amaryllidaceae	
	*Allium dentiferum* Webb et Berthel.
R	*Allium flavum* L. subsp. *flavum*
R	*Allium horvatii* Lovrić
R	*Allium lusitanicum* Lam.
	*Allium sphaerocephalon* L.
	*Allium tenuiflorum* Ten.
	*Allium vineale* L.
	*Galanthus nivalis* L.
	*Narcissus poëticus* L.
Asparagaceae	
	*Loncomelos brevistylus* (Wolfner) Dostál
	*Loncomelos pyrenaicum* (L.) Hrouda ex J. Holub subsp. *pyrenaicum*
	*Muscari neglectum* Guss. ex Ten.
MR	*Ornithogalum comosum* L.
E	*Ornithogalum etruscum* Parl.
	*Ornithogalum umbellatum* L.
	*Polygonatum multiflorum* (L.) All.
	*Scilla bifolia* L
Juncaceae	
	*Juncus articulatus* L. subsp. *articulatus*
	*Juncus bufonius* L.
	*Juncus inflexus* L. subsp. *inflexus*
RR	*Juncus striatus* Schousb. ex E. Mey.
	*Luzula campestris* (L.) DC. subsp. *campestris*
	*Luzula sylvatica* (Huds.) Gaudin subsp. *sylvatica*
Cyperaceae	
	*Carex caryophyllea* Latourr.
	*Carex distans* L.
	*Carex divulsa* Stokes
	*Carex flacca* Schreb. subsp. *flacca*
	*Carex halleriana* Asso
	*Carex hirta* L.
	*Carex kitaibeliana* Degen ex Bech.
R	*Carex leporina* L.
RR	*Carex liparocarpos* Gaudin subsp. *liparocarpos*
	*Carex macrolepis* DC.
	*Carex otrubae* Podp.
R	*Carex pairae* F.W. Schultz
RR	*Carex panicea* L.
	*Carex pendula* Huds.
	*Carex sylvatica* Huds.
R	*Carex viridula* Michx.
	*Schoenoplectus lacustris* (L.) Palla
	*Scirpoides holoschoenus* (L.) Soják
Poaceae	
R	*Agrostis canina* L.
	*Agrostis stolonifera* L.
	*Aira caryophyllea* L. subsp. *caryophyllea*
	*Aira elegantissima* Schur subsp. *elegantissima*
	*Alopecurus aequalis* Sobol.
*	*Alopecurus pratensis* L. subsp. *pratensis*
	*Anisantha sterilis* (L.) Nevski
	*Anisantha tectorum* (L.) Nevski
	*Anthoxanthum odoratum* L. subsp. *odoratum*
	*Arrhenatherum elatius* (L.) P. Beauv. ex J. Presl & C. Presl subsp. *elatius*
RR	*Avenella flexuosa* (L.) Drejer subsp. *flexuosa*
R	*Bellardiochloa variegata* (Lam.) Kerguélen subsp. *variegata*
E – R	*Brachypodium genuense* (DC.) Roem. et Schult.
	*Brachypodium rupestre* (Host) Roem. et Schult.
	*Brachypodium sylvaticum* (Huds.) P.Beauv. subsp. *sylvaticum*
	*Briza media* L.
	*Bromopsis erecta* (Huds.) Fourr.
	*Bromopsis ramosa* (Huds.) Holub subsp. *ramosa*
	*Bromus commutatus* Schrad.
	*Bromus hordeaceus* L. subsp. *hordeaceus*
R	*Bromus lanceolatus* Roth
	*Bromus racemosus* L. subsp. *racemosus*
	*Bromus squarrosus* L. subsp. *squarrosus*
	*Cynodon dactylon* (L.) Pers.
	*Cynosurus cristatus* L.
	*Cynosurus echinatus* L.
	*Dactylis glomerata* L. subsp. *glomerata*
	*Dasypyrum villosum* (L.) P. Candargy
	*Elymus caninus* (L.) L.
	*Elymus repens* (L.) Gould. subsp. *repens*
	*Festuca circummediterranea* Patzke
	*Festuca heterophylla* Lam.
R	*Festuca inops* De Not.
	*Festuca laevigata* Gaudin
	*Festuca stricta* Host subsp. *trachyphylla* (Hack.) Patzke ex Pils
	*Glyceria notata* Chevall.
E	*Helictochloa praetutiana* (Parl. ex Arcang.) Bartolucci, F.Conti, Peruzzi & Banfi subsp. *praetutiana*
	*Holcus lanatus* L. subsp. *lanatus*
R	*Hordelymus europaeus* (L.) Harz
	*Koeleria australis* A. Kern.
	*Koeleria callierii* (Domin) Ujhelyi
E	*Koeleria splendens* C. Presl
	*Koeleria subcaudata* (Asch. & Graebn.) Ujhelyi
	*Leucopoa dimorpha* (Guss.) H.Scholz & Foggi
	*Lolium arundinaceum* (Schreb.) Darbysh. subsp. *arundinaceum*
	*Lolium multiflorum* Lam.
	*Lolium perenne* L.
	*Lolium pratense* (Huds.) Darbysh.
	*Melica ciliata* L. subsp. *ciliata*
	*Melica uniflora* Retz.
	*Nardus stricta* L.
	*Phleum alpinum* L.
	*Phleum nodosum* L.
	*Phleum hirsutum* Honck. subsp. *ambiguum* (Ten.) Cif. & Giacom.
	*Phleum pratense* L. subsp. *pratense*
	*Poa alpina* L. subsp. *alpina*
	*Poa annua* L.
	*Poa bulbosa* L.
	*Poa compressa* L.
	*Poa infirma* Kunth
MR	*Poa molinerii* Balb.
	*Poa nemoralis* L. subsp. *nemoralis*
	*Poa pratensis* L. subsp. *pratensis*
	*Poa sylvicola* Guss.
	*Poa trivialis* L.
	*Sesleria autumnalis* (Scop.) F.W. Schultz
	*Sesleria juncifolia* Suffren subsp. *juncifolia*
E	*Sesleria nitida* Ten. subsp. *nitida*
E	*Stipa dasyvaginata* Martinovský subsp. *apenninicola* Martinovský et Moraldo
	*Trisetaria flavescens* (L.) Baumg. subsp. *flavescens*
E – RR	*Trisetaria villosa* (Bertol.) Banfi et Soldano
Ranunculaceae	
	*Actaea spicata* L.
	*Anemone ranunculoides* (L.) Holub
	*Clematis vitalba* L.
	*Delphinium ajacis* L.
	*Delphinium consolida* L. subsp. *consolida*
	*Delphinium fissum* Waldst. et Kit. subsp. *fissum*
	*Eranthis hyemalis* (L.) Salisb.
	*Ficaria verna Huds.* subsp. *verna*
	*Hepatica nobilis* Schreb.
R	*Ranunculus acris* L. subsp. *acris*
E – R	*Ranunculus apenninus* (Chiov.) Pignatti
MR	*Ranunculus bulbosus* L. subsp. *bulbosus*
R	*Ranunculus gramineus* L.
	*Ranunculus illyricus* L.
	*Ranunculus lanuginosus* L.
	*Ranunculus millefoliatus* Vahl
	*Ranunculus monspeliacus* L. subsp. *monspeliacus*
	*Ranunculus repens* L.
	*Ranunculus sardous* Crantz
	*Ranunculus sceleratus* L.
E – MR	*Ranunculus thomasii* Ten.
MR	*Ranunculus tuberosus* Lapeyr.
Papaveraceae	
	*Corydalis cava* (L.) Schweigg. et Körte subsp. *cava*
	*Corydalis pumila* (Host) Rchb.
E – RR	*Corydalis densiflora* C.Presl subsp. *apennina* F.Conti, Bartolucci & Uzunov
	*Papaver dubium* L. subsp. *dubium*
	*Papaver rhoeas* L. subsp. *rhoeas*
Crassulaceae	
	*Petrosedum rupestre* (L.) P.V.Heath
	*Sedum acre* L.
	*Sedum album* L.
R	*Sedum atratum* L. subsp. *atratum*
	*Sedum dasyphyllum* L.
	*Sedum hispanicum* L.
	*Sedum rubens* L.
	*Sedum sexangulare* L.
R	*Sempervivum arachnoideum* L.
E	*Sempervivum riccii* Iberite et Anzal.
Grossulariaceae	
R	*Ribes alpinum* L.
MR	*Ribes multiflorum* Kit. ex Roem. et Schult. subsp. *multiflorum*
R	*Ribes uva*-*crispa* L. subsp. *uva-crispa*
Saxifragaceae	
	*Saxifraga adscendens* L. subsp. *adscendens*
	*Saxifraga bulbifera* L.
R	*Saxifraga callosa* Sm. subsp. *callosa*
E – R	*Saxifraga exarata* Vill. subsp. *ampullacea* (Ten.) D.A.Webb
	*Saxifraga granulata* L. subsp. *granulata*
	*Saxifraga paniculata* Mill.
E	*Saxifraga porophylla* Bertol. subsp. *porophylla*
	*Saxifraga rotundifolia* L. subsp. *rotundifolia*
	*Saxifraga tridactylites* L.
Fabaceae	
	*Anthyllis montana* L. subsp. *jacquinii* (Rchb.f.) Rohlena
R	*Anthyllis vulneraria* L. subsp. *pulchella* (Vis.) Bornm.
	*Anthyllis vulneraria* L. subsp. *rubriflora* (DC.) Arcang.
	*Astragalus depressus* L. subsp. *depressus*
	*Astragalus glycyphyllos* L.
R	*Astragalus sempervirens* Lam.
E – MR	*Astragalus sirinicus* Ten.
	*Colutea arborescens* L.
	*Coronilla minima* L. subsp. *minima*
	*Coronilla scorpioides* (L.) W.D.J. Koch
	*Cytisophyllum sessilifolium* (L.) O. Lang
	*Cytisus hirsutus* L.
	*Lotus herbaceus* (Vill.) Jauzein
RR	*Genista sagittalis* L.
	*Genista tinctoria* L.
	*Hippocrepis comosa* L. subsp. *comosa*
MR	*Hippocrepis glauca* Ten.
	*Lathyrus cicera* L.
	*Lathyrus latifolius* L.
	*Lathyrus nissolia* L.
	*Lathyrus pratensis* L. subsp. *pratensis*
	*Lathyrus sphaericus* Retz.
	*Lathyrus sylvestris* L. subsp. sylvestris
	*Lathyrus venetus* (Mill.) Wohlf.
	*Lathyrus vernus* (L.) Bernh.
MR	*Lotus corniculatus* L. subsp. *alpinus* (DC.) Rothm.
	*Lotus corniculatus* L. subsp. *corniculatus*
	*Medicago falcata* L. subsp. *falcata*
	*Medicago lupulina* L.
	*Medicago minima* (L.) L.
RR	*Medicago prostrata* Jacq. subsp. *prostrata*
NAT	*Medicago sativa* L.
RR	*Onobrychis arenaria* (Kit.) DC. subsp. *arenaria*
	*Onobrychis viciifolia* Scop.
	*Ononis pusilla* L. subsp. *pusilla*
	*Ononis reclinata* L.
	*Ononis spinosa* L. subsp. *spinosa*
E – RR	*Oxytropis pilosa* (L.) DC. subsp. *caputoi* (Moraldo et La Valva) Brilli-Catt., Di Massimo et Gubellini
	*Securigera varia* (L.) Lassen
	*Spartium junceum* L.
	*Trifolium alpestre* L.
	*Trifolium angustifolium* L. subsp. *angustifolium*
	*Trifolium arvense* L.
	*Trifolium campestre* Schreb.
	*Trifolium dubium* Sibth.
	*Trifolium fragiferum* L. subsp. *fragiferum*
	*Trifolium incarnatum* L. subsp. *molinerii* (Balb. ex Hornem.) Ces.
	*Trifolium micranthum* Viv.
R	*Trifolium montanum* L. subsp. *rupestre* (Ten.) Nyman
	*Trifolium nigrescens* Viv. subsp. *nigrescens*
	*Trifolium ochroleucum* Huds.
	*Trifolium pallidum* Waldst. et Kit.
RR	*Trifolium phleoides* Willd.
	*Trifolium pratense* L. subsp. *pratense*
E	*Trifolium pratense* L. subsp. *semipurpureum* (Strobl) Pignatti
	*Trifolium repens* L.
	*Trifolium scabrum* L.
	*Trifolium squarrosum* L.
R	*Trifolium thalii* Vill.
	*Trigonella gladiata* M. Bieb.
	*Trigonella officinalis* (L.) Coulot & Rabaute
	*Vicia angustifolia* L.
	*Vicia cracca* L.
	*Vicia dasycarpa* Ten.
	*Vicia incana* Gouan
MR	*Vicia onobrychioides* L.
	*Vicia sepium* L.
Polygalaceae	
R	*Polygala alpestris* Rchb. subsp. *alpestris*
	*Polygala major* Jacq.
MR	*Polygala nicaeensis* W.D.J. Koch subsp. *mediterranea* Chodat
Rosaceae	
	*Agrimonia eupatoria* L. subsp. *eupatoria*
RR	*Alchemilla cinerea* Buser
MR	*Alchemilla glaucescens* Wallr.
R	*Alchemilla monticola* Opiz
MR	*Alchemilla strigosula* Buser
	*Amelanchier ovalis* Medik. subsp. *ovalis*
	*Aremonia agrimonoides* (L.) DC. subsp. *agrimonoides*
MR	*Cotoneaster integerrimus* Medik.
	*Crataegus laevigata* (Poir.) DC.
	*Crataegus monogyna* Jacq.
	*Filipendula vulgaris* Moench
	*Fragaria vesca* L. subsp. *Vesca*
MR	*Geum molle* Vis. et Pancic
	*Geum urbanum* L.
NAT	*Malus domestica* (Suckow.) Borkh.
	*Malus sylvestris* (L.) Mill.
R	*Potentilla detommasii* Ten.
	*Potentilla micrantha* Ramond ex DC.
R	*Potentilla pedata* Willd ex Hornem.
	*Potentilla recta* L.subsp. *recta*
	*Potentilla reptans* L.
E	*Potentilla rigoana* Th.Wolf
	*Poterium sanguisorba* subsp. *balearicum* (Bourg. ex Nyman) Stace
	*Prunus avium* (L.) L.
	*Prunus mahaleb* L.
	*Prunus* spinosa L. subsp. *spinosa*
	*Pyrus* communis L. subsp. *pyraster* (L.) Ehrh.
	*Rosa arvensis* Huds.
	*Rosa balsamica* Besser
	*Rosa canina* L. s.s.
	*Rosa corymbifera* Borkh.
MR	*Rosa montana* Chaix
	*Rosa pouzinii* Tratt.
	*Rosa spinosissima* L.
	*Rosa squarrosa* (A. Rau) Boreau
MR	*Rosa villosa* L.
	*Rubus caesius* L.
	*Rubus canescens* DC.
	*Rubus hirtus* Waldst. et Kit.
	*Rubus idaeus* L. subsp. *idaeus*
	*Rubus ulmifolius* Schott
	*Sorbus aria* (L.) Crantz
Rhamnaceae	
R	*Atadinus alpinus* (L.) Raf.
R	*Atadinus fallax* (Boiss.) Hauenschild
R	*Atadinus pumilus* (Turra) Hauenschild subsp. *pumilus*
R	*Rhamnus saxatilis* Jacq.
Urticaceae	
	*Urtica dioica* L. subsp. *dioica*
Fagaceae	
	*Fagus sylvatica* L. subsp. *sylvatica*
	*Quercus cerris* L.
	*Quercus pubescens* Willd. subsp. *pubescens*
Betulaceae	
	*Corylus avellana* L.
	*Ostrya carpinifolia* Scop.
Cucurbitaceae	
	*Bryonia dioica* Jacq.
Celastraceae	
	*Euonymus europaeus* L.
	*Euonymus latifolius* (L.) Mill.
RR	*Parnassia palustris* L. subsp. *palustris*
Violaceae	
	*Viola alba* Besser subsp. *dehnhardtii* (Ten.) W.Becker
	*Viola arvensis* Murray
E	*Viola eugeniae* Parl. subsp. *eugeniae*
MR	*Viola kitaibeliana* Schult.
	*Viola odorata* L.
	*Viola reichenbachiana* Jord. ex Boreau
Salicaceae	
	*Populus tremula* L.
	*Salix apennina* A.K. Skortsov
	*Salix caprea* L.
	*Salix eleagnos* Scop.
MR	*Salix purpurea* L. subsp. *purpurea*
Linaceae	
	*Linum catharticum* L. subsp. *catharticum*
	*Linum corymbulosum* Rchb.
	*Linum strictum* L.
	*Linum tenuifolium* L.
MR	*Linum tommasinii* (Rchb.) Nyman
	*Linum trigynum* L.
	*Linum usitatissimum* L. subsp. *angustifolium* (Huds.) Thell.
R	*Linum viscosum* L.
Hypericaceae	
RR	*Hypericum hyssopifolium* Chaix
	*Hypericum perforatum* L. subsp. *veronense* (Schrank) Ces.
	*Hypericum tetrapterum* Fr.
Euphorbiaceae	
	*Euphorbia amygdaloides* L.
	*Euphorbia cyparissias* L.
	*Euphorbia falcata* L. subsp. *falcata*
R	*Euphorbia myrsinites* L. subsp. *myrsinites*
Geraniaceae	
	*Erodium ciconium* (L.) L’Hér.
	*Erodium cicutarium* (L.) L’Hér. subsp. *cicutarium*
	*Geranium columbinum* L.
	*Geranium dissectum* L.
	*Geranium lucidum* L.
	*Geranium molle* L.
MR	*Geranium nodosum* L.
	*Geranium purpureum* Vill.
	*Geranium pyrenaicum* Burm. fil. subsp. *pyrenaicum*
	*Geranium robertianum* L.
	*Geranium rotundifolium* L.
	*Geranium sanguineum* L.
Onagraceae	
	*Chamaenerion angustifolium* (L.) Scop.
	*Chamaenerion dodonaei* (Vill.) Schur ex Fuss
	*Epilobium montanum* L.
	*Epilobium parviflorum* Schreb.
Lythraceae	
	*Lythrum hyssopifolia* L.
Sapindaceae	
	*Acer campestre* L.
	*Acer opalus* Mill. subsp. *obtusatum* (Waldst. & Kit. ex Willd.) Gams
	*Acer pseudoplatanus* L.
Thymelaeaceae	
	*Daphne laureola* L.
	*Daphne oleoides* Schreb. subsp. *oleoides*
Cistaceae	
	*Helianthemum apenninum* (L.) Mill. subsp. *apenninum*
MR	*Helianthemum nummularium* (L.) Mill. subsp. *grandiflorum* (Scop.) Schinz et Thell.
	*Helianthemum nummularium* (L.) Mill. subsp. *obscurum* (Celak.) Holub
	*Helianthemum oelandicum* (L.) Dum. Cours. subsp. *incanum* (Willk.) G. López
	*Helianthemum salicifolium* (L.) Mill.
Malvaceae	
	*Malva alcea* L.
	*Malva sylvestris* L.
	*Tilia platyphyllos* Scop. subsp. *platyphyllos*
Resedaceae	
	*Reseda luteola* L.
Brassicaceae	
	*Aethionema saxatile* (L.) R.Br.
	*Alliaria petiolata* (M. Bieb.) Cavara et Grande
	*Alyssum alyssoides* (L.) L.
	*Alyssum simplex* Rudolphi
	*Arabidopsis thaliana* (L.) Heynh.
	*Arabis alpina* L. subsp. *caucasica* (Willd.) Briq.
RR	*Arabis auriculata* Lam.
	*Arabis collina* Ten. subsp. *collina*
	*Arabis hirsuta* (L.) Scop.
	*Arabis sagittata* (Bertol.) DC.
	*Barbarea bracteosa* Guss.
	*Barbarea vulgaris* R. Br.
	*Biscutella laevigata* L. subsp. *laevigata* var. *laevigata*
	*Bunias erucago* L.
	*Capsella bursa*-*pastoris* (L.) Medik. subsp. *bursa*-*pastoris*
	*Capsella rubella* Reut.
	*Cardamine bulbifera* (L.) Crantz
	*Cardamine impatiens* L. subsp. *impatiens*
RR	*Conringia austriaca* (Jacq.) Sweet
	*Draba aizoides* L. subsp. *aizoides*
	*Draba verna* L. subsp. *verna*
E – RR	*Erysimum majellense* Polatschek – after Iocchi et al. (2010) this is the second record for Lazio, thus representing a confirmation of its presence in the region.
E	*Erysimum pseudorhaeticum* Polatschek
R	*Hesperis laciniata* All. subsp. *laciniata*
	*Hesperis matronalis* L. subsp. *matronalis*
	*Hornungia petraea* (L.) Rchb. subsp. *petraea*
	*Isatis tinctoria* L. subsp. *tinctoria*
	*Microthlaspi perfoliatum* (L.) F.K.Mey.
	*Mummenhoffia alliacea* (L.) Esmailbegi & Al-Shehbaz
	*Pseudoturritis turrita* (L.) Al-Shehbaz
	*Rapistrum rugosum* (L.) All.
	*Sinapis arvensis* L. subsp. *arvensis*
MR	*Turritis glabra* L.
Loranthaceae	
	*Loranthus europaeus* Jacq.
Santalaceae	
	*Thesium humifusum* DC.
R	*Thesium linophyllon* L.
R	*Viscum album* L.
Plumbaginaceae	
E	*Armeria canescens* (Host) Ebel
Polygonaceae	
R	*Bistorta officinalis* Delarbre
	*Fallopia convolvulus* (L.) A. Löve
	*Polygonum arenastrum* Boreau subsp. *arenastrum*
	*Polygonum aviculare* L. subsp. *aviculare*
	*Rumex acetosa* L. subsp. *acetosa*
	*Rumex acetosella* L. subsp. *pyrenaicus* (Pourr. ex Lapeyr.) Akeroyd
R	*Rumex alpinus* L.
R	*Rumex arifolius* All.
	*Rumex crispus* L.
	*Rumex scutatus* L. subsp. *scutatus*
Caryophyllaceae	
	*Agrostemma githago* L. subsp. *githago*
	*Arenaria leptoclados* (Rchb.) Guss. subsp. *leptoclados*
	*Arenaria serpyllifolia* L. subsp. *serpyllifolia*
	*Cerastium arvense* L. subsp. *arvense*
	*Cerastium brachypetalum* Desp. ex Pers. subsp. *brachypetalum*
	*Cerastium brachypetalum Desp.* ex Pers. subsp. *roeseri* (Boiss. et Heldr.) Nyman
	*Cerastium brachypetalum* Desp. ex Pers. subsp. *tenoreanum* (Ser.) Soó
	*Cerastium ligusticum* Viv.
E	*Cerastium tomentosum* L.
	*Dianthus carthusianorum* L. subsp. *carthusianorum*
	*Dianthus deltoides* L. subsp. *deltoides*
R	*Dianthus hyssopyfolius* L.
	*Dianthus longicaulis* Ten.
R	*Drypis spinosa* L. subsp. *spinosa*
RR	*Herniaria glabra* L. subsp. *nebrodensis* Nyman
	*Herniaria hirsuta* L. subsp. *hirsuta*
	*Herniaria incana* Lam.
RR	*Mcneillia graminifolia* (Ard.) Dillenb. & Kadereit subsp. *clandestina* (Port.) Dillenb. & Kadereit
	*Moehringia trinervia* (L.) Clairv.
	*Paronychia kapela* (Hacq.) A. Kern. subsp. *kapela*
	*Petrorhagia dubia* (Raf.) G. López et Romo
	*Petrorhagia prolifera* (L.) P. W. Ball. et Heywood
	*Petrorhagia saxifraga* (L.) Link subsp. *saxifraga*
	*Rabelera holostea* (L.) M.T.Sharples & E.A.Tripp
	*Sabulina glaucina* (Dvořáková) Dillenb. & Kadereit
	*Sabulina tenuifolia* (L.) Rchb. subsp. *tenuifolia*
R	*Sabulina verna* (L.) Rchb. subsp. *verna*
	*Sagina alexandrae* Iamonico
	*Saponaria ocymoides* L.
	*Scleranthus annuus* L.
	*Scleranthus polycarpos* L.
MR	*Silene ciliata* Pourr. subsp. *graefferi* (Guss.) Nyman
	*Silene conica* L.
	*Silene dioica* (L.) Clairv.
	*Silene italica* (L.) Pers. subsp. *italica*
	*Silene latifolia* Poir.
R	*Silene multicaulis* Guss. subsp. *multicaulis*
	*Silene nemoralis* Waldst. et Kit.
E – MR	*Silene notarisii* Ces.
MR	*Silene nutans* L. subsp. *nutans*
	*Silene otites* (L.) Wibel
MR	*Silene saxifraga* L.
	*Silene vulgaris* (Moench) Garcke subsp. *vulgaris*
	*Stellaria media* (L.) Vill. subsp. *media*
	*Stellaria nemorun* L. subsp. *montana* (Pierrat) Berher
	*Stellaria pallida* (Dumort.) Crép.
Chenopodiaceae	
	*Blitum bonus-henricus* (L.) Rchb.
	*Chenopodium album* L.
	*Chenopodium opulifolium* Schrad. ex W.D.J. Koch et Ziz
	*Chenopodium vulvaria* L.
Primulaceae	
	*Cyclamen hederifolium* Aiton subsp. *hederifolium*
	*Lysimachia arvensis* (L.) U.Manns & Anderb. subsp. *arvensis*
R	*Primula veris* L. subsp. *columnae* (Ten.) Maire & Petitm.
	*Primula vulgaris* Huds. subsp. *vulgaris*
Ericaceae	
	*Monotropa hypopitys* L.
	*Orthilia secunda* (L.) House
R	*Vaccinium myrtillus* L.
Rubiaceae	
R	*Asperula aristata* L.f. subsp. *scabra* Nyman
	*Asperula cynanchica* L.
	*Asperula purpurea* (L.) Ehrend.
	*Cruciata glabra* (L.) C.Bauhin ex Opiz
	*Cruciata laevipes* Opiz
MR	*Cruciata pedemontana* (Bellardi) Ehrend.
	*Galium album* Mill. subsp. *album*
	*Galium aparine* L.
	*Galium corrudifolium* Vill.
	*Galium lucidum* All.
E – MR	*Galium magellense* Ten.
	*Galium mollugo* L.
	*Galium odoratum* (L.) Scop.
	*Galium verum* L. subsp. *verum*
	*Sherardia arvensis* L.
Gentianaceae	
	*Centaurium pulchellum* (Sw.) Druce subsp. *pulchellum*
	*Gentiana cruciata* L. subsp. *cruciata*
	*Gentiana lutea* L. subsp. *lutea*
MR	*Gentiana utriculosa* L.
	*Gentiana verna* L. subsp. *verna*
E	*Gentianella columnae* (Ten.) Holub
Apocynaceae	
	*Vincetoxicum hirundinaria* Medik. subsp. *hirundinaria*
Boraginaceae	
	*Aegonychon purpurocaeruleum* (L.) Holub
	*Anchusa azurea* Mill.
	*Buglossoides arvensis* (L.) I.M. Johnst.
MR	*Buglossoides incrassata* (Guss.) I.M. Johnst. subsp. *incrassata*
E	*Cynoglossum apenninum* L.
E – R	*Cynoglossum magellense* Ten.
	*Cynoglossum montanum* L.
	*Cynoglossum officinale* L.
	*Cynoglottis barrelieri* (All.) Vural et Kit Tan subsp. *barrelieri*
	*Echium italicum* L. subsp. *italicum*
	*Echium vulgare* L.
R	*Myosotis alpestris* F.W.Schmidt
	*Myosotis arvensis* (L.) Hill subsp. *arvensis*
E	*Myosotis decumbens* Host subsp. *florentina* Grau
MR	*Myosotis graui* Selvi
	*Onosma echioides* (L.) L.
E	*Pulmonaria vallarsae* A.Kern. subsp. *apennina* (Cristof. & Puppi) L.Cecchi & Selvi
Convolvulaceae	
	*Convolvulus arvensis* L.
	*Cuscuta epithymum* (L.) L. subsp. *epithymum*
	*Cuscuta planiflora* Ten.
Plantaginaceae	
	*Chaenorhinum minus* (L.) Lange subsp. *minus*
	*Digitalis ferruginea* L.
E	*Digitalis micrantha* Roth ex Schweigg.
	*Globularia bisnagarica* L.
	*Globularia meridionalis* (Podp.) O.Schwarz
E	*Linaria purpurea* (L.) Mill.
	*Plantago argentea* Chaix
	*Plantago atrata* Hoppe subsp. *atrata*
	*Plantago lanceolata* L.
	*Plantago lanceolata* L. var. *sphaerostachya* Mert. et W.D.J. Koch
	*Plantago major* L. subsp. *major*
	*Plantago media* L. subsp. *media*
	*Plantago sempervirens* Crantz
	*Plantago subulata* L.
	*Veronica anagallis*-*aquatica* L. subsp. *anagallis*-*aquatica*
	*Veronica arvensis* L.
MR	*Veronica barrelieri* H.Schott ex Roem. & Schult. subsp. *barrelieri*
	*Veronica beccabunga* L. subsp. *beccabunga*
	*Veronica chamaedrys* L. subsp. *chamaedrys*
	*Veronica cymbalaria* Bodard subsp. *cymbalaria*
	*Veronica hederifolia* L. subsp. *hederifolia*
R	*Veronica orsiniana* Ten. subsp. *orsiniana*
NAT	*Veronica persica* Poir.
	*Veronica polita* Fr.
MR	*Veronica prostrata* L.
	*Veronica serpyllifolia* L.
Scrophulariaceae	
	*Scrophularia auriculata* L. subsp. *auriculata*
	*Scrophularia canina* L.
R	*Scrophularia juratensis* Schleich.
	*Scrophularia nodosa* L.
	*Scrophularia scopolii* Hoppe ex Pers.
	*Scrophularia vernalis* L.
	*Verbascum longifolium* Ten.
	*Verbascum mallophorum* Boiss. et Heldr.
	*Verbascum pulverulentum* Vill.
Lamiaceae	
	*Ajuga chamaepitys* (L.) Schreb. subsp. *chamaepitys*
	*Ajuga reptans* L.
E – MR	*Betonica alopecuros L.* subsp. *divulsa* (Ten.) Bartolucci & Peruzzi
	*Clinopodium menthifolium* (Host) Merino subsp. *menthifolium*
	*Clinopodium vulgare* L. subsp. *vulgare*
	*Galeopsis angustifolia* Hoffm. subsp. *angustifolia*
	*Lamium bifidum* Cirillo subsp. *bifidum*
	*Lamium garganicum* L. subsp. *laevigatum* Arcang.
	*Lamium maculatum* L.
	*Lamium purpureum* L.
	*Marrubium incanum* Desr.
	*Melittis melissophyllum* L. subsp. *melissophyllum*
	*Mentha longifolia* (L.) L.
MR	*Mentha microphylla* C. Koch
	*Origanum vulgare* L. subsp. *vulgare*
	*Prunella laciniata* (L.) L.
	*Prunella vulgaris* L. subsp. *vulgaris*
	*Salvia glutinosa* L.
	*Salvia pratensis* L.
	*Salvia verbenaca* L.
	*Salvia virgata* Jacq.
	*Stachys heraclea* All.
	*Stachys recta* L. subsp. *recta*
R	*Stachys recta* L. subsp. *subcrenata* (Vis.) Briq.
	*Stachys tymphaea* Hausskn.
	*Teucrium chamaedrys* L. subsp. *chamaedrys*
	*Teucrium montanum* L.
	*Thymus longicaulis* C.Presl subsp. *longicaulis*
RR	*Thymus oenipontanus* Heinr.Braun ex Borbás
R	*Thymus praecox* Opiz subsp. *polytrichus* (Borbás) Jalas
	*Thymus striatus* Vahl
	*Ziziphora acinos* (L.) Melnikov
	*Ziziphora granatensis* (Boiss. & Reut.) Melnikov subsp. *alpina* (L.) Bräuchler & Gutermann
Orobanchaceae	
MR	*Euphrasia liburnica* Wettst.
	*Euphrasia stricta* D. Wolff ex J.F. Lehm.
	*Orobanche artemisiae-campestris* Gaudin
	*Orobanche caryophyllacea* Sm.
	*Orobanche gracilis* Sm.
	*Orobanche minor* Sm.
MR	*Orobanche reticulata* Wallr. subsp. *reticulata*
R	*Orobanche teucrii* Holandre
R	*Orobanche variegata* Wallr.
	*Parentucellia latifolia* (L.) Caruel
	*Pedicularis comosa* L. subsp. *comosa*
E	*Pedicularis elegans* Ten.
MR	*Pedicularis tuberosa* L.
	*Rhinanthus alectorolophus* (Scop.) Pollich
	*Rhinanthus minor* L.
E	*Rhinanthus wettsteinii* (Sterneck) Soó
Campanulaceae	
	*Campanula glomerata* L. subsp. *glomerata*
E – MR	*Campanula micrantha* Bertol.
	*Campanula rapunculus* L.
	*Campanula scheuchzeri* Vill. subsp. *scheuchzeri*
E	*Campanula tanfanii* Podlech
	*Campanula trachelium* L. subsp. *trachelium*
	*Edraianthus graminifolius* (L.) A. DC. subsp. *graminifolius*
	*Legousia hybrida* (L.) Delarbre
	*Legousia speculum*-*veneris* (L.) Chaix subsp. *speculum*-*veneris*
R	*Phyteuma orbiculare* L.
Asteraceae	
	*Achillea setacea* Waldst. et Kit. subsp. *setacea*
RR	*Achillea tomentosa* L.
	*Adenostyles alpina* (L.) Bluff & Fingerh. subsp. *alpina*
	*Anthemis arvensis* L. subsp. *arvensis*
R	*Anthemis cretica* L. subsp. *columnae* (Ten.) Franzén
	*Arctium lappa* L.
	*Arctium minus* (Hill) Bernh.
	*Bellis perennis* L.
	*Bellis sylvestris* Cirillo
	*Bombycilaena erecta* (L.) Smoljan
	*Carduus defloratus* L. subsp. *carlinifolius* (Lam.) Ces.
	*Carduus nutans* L. subsp. *nutans*
E	*Carduus nutans* L. subsp. *perspinosus* (Fiori) Arènes
	*Carlina acanthifolia* All. subsp. *acanthifolia*
	*Carlina acaulis* L. subsp. *caulescens* (Lam.) Schübl. et G. Martens
	*Carlina corymbosa* L.
	*Carlina vulgaris* L. subsp. *vulgaris*
	*Carthamus lanatus* L. subsp. *lanatus*
E – MR	*Centaurea ambigua* Guss. subsp. *ambigua*
E	*Centaurea ambigua* Guss. subsp. *nigra* (Fiori) Pignatti
RR	*Centaurea arachnoidea* subsp. *adonidifolia* (Rchb.) F. Conti, Moraldo & Ricceri
	*Centaurea deusta* Ten.
	*Centaurea jacea* L. subsp. *gaudini* (Boiss. et Reut.) Gremli
R	*Centaurea scabiosa* L.
	*Centaurea triumfettii* All.
	*Chondrilla juncea* L.
	*Cichorium intybus* L. subsp. *intybus*
	*Cirsium arvense* (L.) Scop.
	*Cirsium creticum* (Lam.) d’Urv. subsp. *triumfetti* (Lacaita) Werner
	*Cirsium eriophorum* (L.) Scop.
R	*Cirsium palustre* (L.) Scop.
	*Cota segetalis* (Ten.) Holub
	*Cota tinctoria* (L.) J. Gay subsp. *australis* (R. Fern.) Oberprieler et Greuter
	*Cota tinctoria* (L.) J. Gay subsp. *tinctoria*
MR	*Crepis biennis* L.
	*Crepis foetida* L.
E	*Crepis lacera* Ten.
	*Crepis neglecta* L.
	*Crepis vesicaria* L. subsp. *vesicaria*
	*Crupina vulgaris* Cass.
	*Doronicum columnae* Ten.
	*Echinops sphaerocephalus* L. subsp. *sphaerocephalus*
	*Erigeron acris* L. subsp. *acris*
	*Erigeron epiroticus* (Vierh.) Halácsy
	*Eupatorium cannabinum* L. subsp. *cannabinum*
	*Helminthotheca echioides* (L.) Holub
R	*Hieracium bifidum* Kit. ex Hornem.
*	*Hieracium bupleuroides* C.C. Gmel.
RR	*Hieracium cymosum* L. subsp. *cymosum*
E – RR	*Hieracium hoppeanum* Schult.
R	*Hieracium lachenalii* C.C.Gmel.
	*Hieracium murorum* L.
MR	*Hieracium prenanthoides* Vill.
	*Hieracium racemosum* Waldst. et Kit. ex Willd.
RR	*Hieracium tomentosum* (L.) L.
MR	*Hieracium villosum* Jacq.
	*Jacobaea erucifolia* (L.) G.Gaertn., B.Mey. & Scherb. subsp. *erucifolia*
E – R	*Klasea flavescens* (L.) Holub subsp. *cichoracea* (L.) Greuter et Wagenitz
	*Klasea nudicaulis* (L.) Fourr.
	*Lactuca perennis* L. subsp. *perennis*
	*Lactuca sativa* L. subsp. *serriola* (L.) Galasso, Banfi, Bartolucci & Ardenghi
	*Lactuca viminea* (L.) J. Presl et C. Presl subsp. *chondrilliflora* (Boreau) St.-Lag.
	*Lapsana communis* L. subsp. *communis*
	*Leontodon crispus* Vill.
	*Leontodon hispidus* L.
	*Leontodon rosanoi* (Ten.) DC.
	*Leontodon tuberosus* L.
	*Leucanthemum vulgare* Lam. subsp. *vulgare*
NAT	*Matricaria discoidea* DC.
	*Mycelis muralis* (L.) Dumort. subsp. *muralis*
	*Onopordum acanthium* L. subsp. *acanthium*
	*Onopordum illyricum* L. subsp. *illyricum*
	*Pentanema montanum* (L.) D.Gut.Larr., Santos-Vicente, Anderb., E.Rico & M.M.Mart.Ort.
	*Pentanema squarrosum* (L.) D.Gut.Larr., Santos- Vicente, Anderb., E.Rico & M.M.Mart.Ort.
	*Petasites hybridus* (L.) P. Gaertn., B. Mey. et Scherb. subsp. *hybridus*
	*Picris hieracioides* L. subsp. *hieracioides*
	*Pilosella officinarum* Vaill.
	*Pilosella piloselloides* (Vill.) Soják
RR	*Pilosella piloselloides* (Vill.) Soják subsp. *praealta* (Vill. ex Gochnat) S.Bräut. & Greuter
	*Prenanthes purpurea* L.
	*Pseudopodospermum hispanicum* (L.) Zaika, Sukhor. & N.Kilian
	*Ptilostemon strictus* (Ten.) Greuter
	*Pulicaria dysenterica* (L.) Bernh.
	*Scorzonera cana* (C.A.Mey.) Griseb.
RR	*Scorzonera laciniata* L.
RR	*Scorzonera purpurea* L. subsp. *purpurea*
	*Scorzoneroides cichoriacea* (Ten.) Greuter
MR	*Senecio apenninus* Tausch
	*Senecio scopolii* Hoppe et Hornsch. ex Bluff et Fingerh.
R	*Serratula tinctoria* L. subsp. *tinctoria* var. *tinctoria*
	*Solidago virgaurea* L. subsp. *virgaurea*
	*Sonchus asper* (L.) Hill subsp. *asper*
R	*Sonchus asper* (L.) Hill subsp. *glaucescens* (Jord.) Ball
R	*Tanacetum corymbosum* (L.) Sch. Bip. var. *corymbosum*
	*Tanacetum corymbosum* (L.) Sch. Bip. var. *tenuifolium* (Willd.) Briq. et Cavill.
	*Tanacetum parthenium* (L.) Sch. Bip.
	*Taraxacum fulvum* gr.
	*Taraxacum minimum* (V.Brig.) N.Terracc.
	*Taraxacum officinale* Weber
R	*Tragopogon crocifolius* L.
R	*Tragopogon dubius* Scop.
	*Tragopogon porrifolius* L.
	*Tragopogon pratensis* L.
	*Tragopogon samaritani* Heldr. et Sartori ex Boiss.
	*Tussilago farfara* L.
	*Xeranthemum cylindraceum* Sm.
	*Xeranthemum inapertum* (L.) Mill.
Viburnaceae	
	*Adoxa moschatellina* L. subsp. *moschatellina*
	*Sambucus ebulus* L.
	*Sambucus nigra* L.
Dipsacaceae	
	*Dipsacus fullonum* L.
	*Knautia purpurea* (Vill.) Borbás
	*Scabiosa columbaria* L.
Caprifoliaceae	
	*Lonicera alpigena* L. subsp. *alpigena*
	*Lonicera etrusca* Santi
Valerianaceae	
	*Centranthus ruber* (L.) DC. subsp. *ruber*
	*Valeriana officinalis* L.
	*Valeriana tuberosa* L.
	*Valerianella carinata* Loisel.
	*Valerianella eriocarpa* Desv.
	*Valerianella locusta* (L.) Laterr.
Araliaceae	
	*Hedera helix* L. subsp. *helix*
Apiaceae	
	*Aegopodium podagraria* L.
	*Angelica sylvestris* L. subsp. *sylvestris*
	*Anthriscus sylvestris* (L.) Hoffm. subsp. *sylvestris*
	*Bunium bulbocastanum* L.
	*Bupleurum baldense* Turra
	*Bupleurum falcatum* L. subsp. *cernuum* (Ten.) Arcang.
	*Chaerophyllum aureum* L.
MR	*Chaerophyllum hirsutum* L.
	*Chaerophyllum temulum* L.
	*Daucus carota* L. subsp. *carota*
	*Eryngium amethystinum* L.
R	*Heracleum sibiricum* L. subsp. *sibiricum*
R	*Heracleum sibiricum* L. subsp. *ternatum* (Velen.) Briq.
	*Katapsuxis silaifolia* (Jacq.) Reduron, Charpin & Pimenov
	*Oenanthe pimpinelloides* L.
	*Opopanax chironium* (L.) W.D.J. Koch
	*Orlaya grandiflora* (L.) Hoffm.
R	*Pimpinella major* (L.) Huds.
	*Pimpinella saxifraga* L.
	*Pimpinella tragium* Vill.
	*Sanicula europaea* L.
	*Scandix macrorhyncha* C.A.Mey.
	*Scandix pecten*-*veneris* L. subsp. *pecten*-*veneris*
	*Seseli montanum* L. subsp. *montanum*
E	*Siler montanum* Crantz subsp. *siculum* (Spreng.) Iamonico, Bartolucci & F.Conti
	*Tordylium maximum* L.
	*Torilis arvensis* (Huds.) Link subsp. *arvensis*
	*Trinia dalechampii* (Ten.) Janch.
*RR	*Trinia glauca* (L.) Dumort. subsp. *glauca*

## Supplementary materials

Supplementary material 1 Phytosociological releves surveyed in the study area and their syntaxonomical classification Data type Table Explanation note Table with the phytosociological releves surveyed in the study area and their syntaxonomical classification. https://binary.pensoft.net/file/550125This dataset is made available under the Open Database License (http://opendatacommons.org/licenses/odbl/1.0/). The Open Database License (ODbL) is a license agreement intended to allow users to freely share, modify, and use this Dataset while maintaining this same freedom for others, provided that the original source and author(s) are credited.Edda Lattanzi, Eva Del Vico, Roberto Tranquilli, Emmanuele Farris, Michela Marignani, Leonardo Rosati
